# Sequence characteristics, genetic diversity and phylogenetic analysis of the *Cucurbita ficifolia* (Cucurbitaceae) chloroplasts genome

**DOI:** 10.1186/s12864-024-10278-2

**Published:** 2024-04-18

**Authors:** Shuilian He, Bin Xu, Siyun Chen, Gengyun Li, Jie Zhang, Junqiang Xu, Hang Wu, Xuejiao Li, Zhengan Yang

**Affiliations:** 1 College of Landscape and Horticulture, Yunnan Agricultural University, 650201 Kunming, Yunnan, China; 2 Key Laboratory of Vegetable Biology of Yunnan Province, College of Landscape and Horticulture, Yunnan Agricultural University, 650201 Kunming, Yunnan, China; 3 Plant Germplasm and Genomics Center, Germplasm Bank of Wild Species, Kunming Institute of Botany, Chinese Academy of Sciences, 650201 Kunming, Yunnan, China

**Keywords:** *Cucurbita ficifolia*, Genetic diversity, Chloroplast genome, Phylogenetic analysis

## Abstract

**Background:**

*Curcubita ficifolia* Bouché (Cucurbitaceae) has high value as a food crop and medicinal plant, and also has horticultural value as rootstock for other melon species. China is home to many different cultivars, but the genetic diversity of these resources and the evolutionary relationships among them, as well as the differences between *C. ficifolia* and other *Cucurbita* species, remain unclear.

**Results:**

We investigated the chloroplast (cp) genomes of 160 *C. ficifolia* individuals from 31 populations in Yunnan, a major *C. ficifolia* production area in China. We found that the cp genome of *C. ficifolia* is ~151 kb and contains 128 genes, of which 86 are protein coding genes, 34 encode tRNA, and eight encode rRNAs. We also identified 64 SSRs, mainly AT repeats. The cp genome was found to contain a total of 204 SNP and 57 indels, and a total of 21 haplotypes were found in the 160 study individuals. The reverse repeat (IR) region of *C. ficifolia* contained a few differences compared with this region in the six other *Cucurbita* species. Sequence difference analysis demonstrated that most of the variable regions were concentrated in the single copy (SC) region. Moreover, the sequences of the coding regions were found to be more similar among species than those of the non-coding regions. The phylogenies reconstructed from the cp genomes of 61 representative species of Cucurbitaceae reflected the currently accepted classification, in which *C. ficifolia* is sister to the other *Cucurbita* species, however, different interspecific relationships were found between *Cucurbita* species.

**Conclusions:**

These results will be valuable in the classification of *C. ficifolia* genetic resources and will contribute to our understanding of evolutionary relationships within the genus *Cucurbita*.

**Supplementary Information:**

The online version contains supplementary material available at 10.1186/s12864-024-10278-2.

## Introduction

The genus *Cucurbita* (Cucurbitaceae) is thought to have an American origin [1], and comprises 20–27 species [2], the majority of which are herbaceous. Five species are cultivated: *C. argyrosperma*, *C. maxima*, *C. moschata*, *C. ficifolia* and *C. pepo*; these are popular, economically important crops (gourds, squashes and pumpkins) and are widely cultivated in almost all regions having arable land. The genetic diversity and germplasm resources in four of these species (*C. moschata*, *C. argyrosperma, C. maxima*, *and C. pepo*) have been studied in some depth [3–6]. Furthermore, DNA barcoding [2], AFLP [7] and simple sequence repeats (SSRs) markers [8–10] have been developed for the study of genetic diversity and phylogenetic relationships in and between various *Cucurbita* species.

*Cucubita ficifolia* is a short-day plant. It is sensitive to temperature and is not heat-resistant. The plant is known as “black seed squash” in English, and is called “Black Seeded figleaf squash” in Chinese [11]. *C. ficifolia* originated in the Central-South American region [12], and is now grown world-wide in low-latitude/high-altitude regions. There are therefore no wild populations of *C. ficifolia* in China, although this species has a long history of cultivation in Yunnan, Sichuan, Guizhou and other higher altitude regions in the country [13]. Much of Yunnan Province has a climate suitable for the growth and cultivation of *C. ficifolia*, and this province has become the main production area in China. *C. ficifolia* is not as popular as a human food crop as other cultivated *Cucurbita* species, and has therefore received less research attention. However, *C. ficifolia* fruit yields are high, and the plants are strong, will grow on barren ground, and are resistant to cold, drought, and several diseases including *Fusarium* wilt. The species is therefore an important germplasm resource for the breeding of melon cultivars [7]. However, there have been only a few studies into the genetics of this species to date. Therefore, in order to effectively utilize and develop this resource, a better understanding of the genetic diversity of *C. ficifolia*, its differences from other *Cucurbita* species and the phylogenetic relationships within this group are required.

The chloroplast (cp) [14, 15] is important in various plant cell functions, including carbon fixation and photosynthesis as well as the stress response. The cp is semi-autonomous, having a semi-independent genome and encoding its own genetic system for the transcription translation and replication of DNA and RNA [16]. The structure of the cp genome is conserved. The double-stranded, circular DNA molecule has a quadripartite structure, comprising large (LSC) and small (SSC) single-copy regions usually separated by two inverted repeat (IR) regions [17–19]. The composition and order of the cp genes is highly conserved in most angiosperms [20], and the genome ranges between 120 and 160 kb in size [21]. Between 110 and 130 genes are usually present on the cp genome in flowering plants [22], and comprise genes related to photosynthesis, transcription/translation, and biosynthesis. Throughout the evolutionary history of the chloroplast, the cp genome has undergone certain major alterations, including the loss of specific introns, large-scale genomic rearrangements, and IR region contraction and expansion.

The cp genome is inherited from the maternal parent, is relatively small and simple in structure, with a low molecular weight and nucleotide substitution rate [23, 24]. The first cp genome to be assembled was that of *Nicotiana tabacum* [25], and with the rise of sequencing technology, the cp genomes of many plant species have been sequenced. Cp genomes, which are often used in species identification and analyses of genetic diversity [26–28], as well as phylogenetic, taxonomic and evolutionary studies [29] have even been called “DNA super barcodes” [23].

However, although the sequence and gene content of the plant cp genome are highly conserved [30], sequence variation occurs through loss or mutation of genes and pseudogenization [31]. These variants are valuable in species comparisons in the study of evolutionary relationships between taxa and plant taxonomy [32, 33].

We previously reported the cp genome from a single individual of *C. ficifolia* [34]. However, the genetic diversity in the cp genome in *C. ficifolia* resources from different regions are unknown. Compared with other cultivated melon species, *C. ficifolia* has received little research attention, and its systematic placement and relationships with other melons and gourds is unclear. In this study, we collected samples from 160 *C. ficifolia* landraces from Yunnan Province, China, and the cp genomes from these landraces were sequenced and assembled. In addition, we analyzed the GC content of the *C. ficifolia* cp genome, as well as the number of genes and repeat sequences, the codon usage bias and simple sequence repeats (SSR), and compared the IR region and gene differentiation in *C. ficifolia* with those in other *Cucurbita* species. The evolutionary relationships between *C. ficifolia* and other Cucurbitaceae species as inferred from their cp genomes is also discussed. These results will inform the wider utilization of *C. ficifolia* germplasm resources and further our knowledge of the evolution of this important family.

## Materials and methods

### Plant materials, DNA extraction and whole genome resequencing

**Fig. 1:**
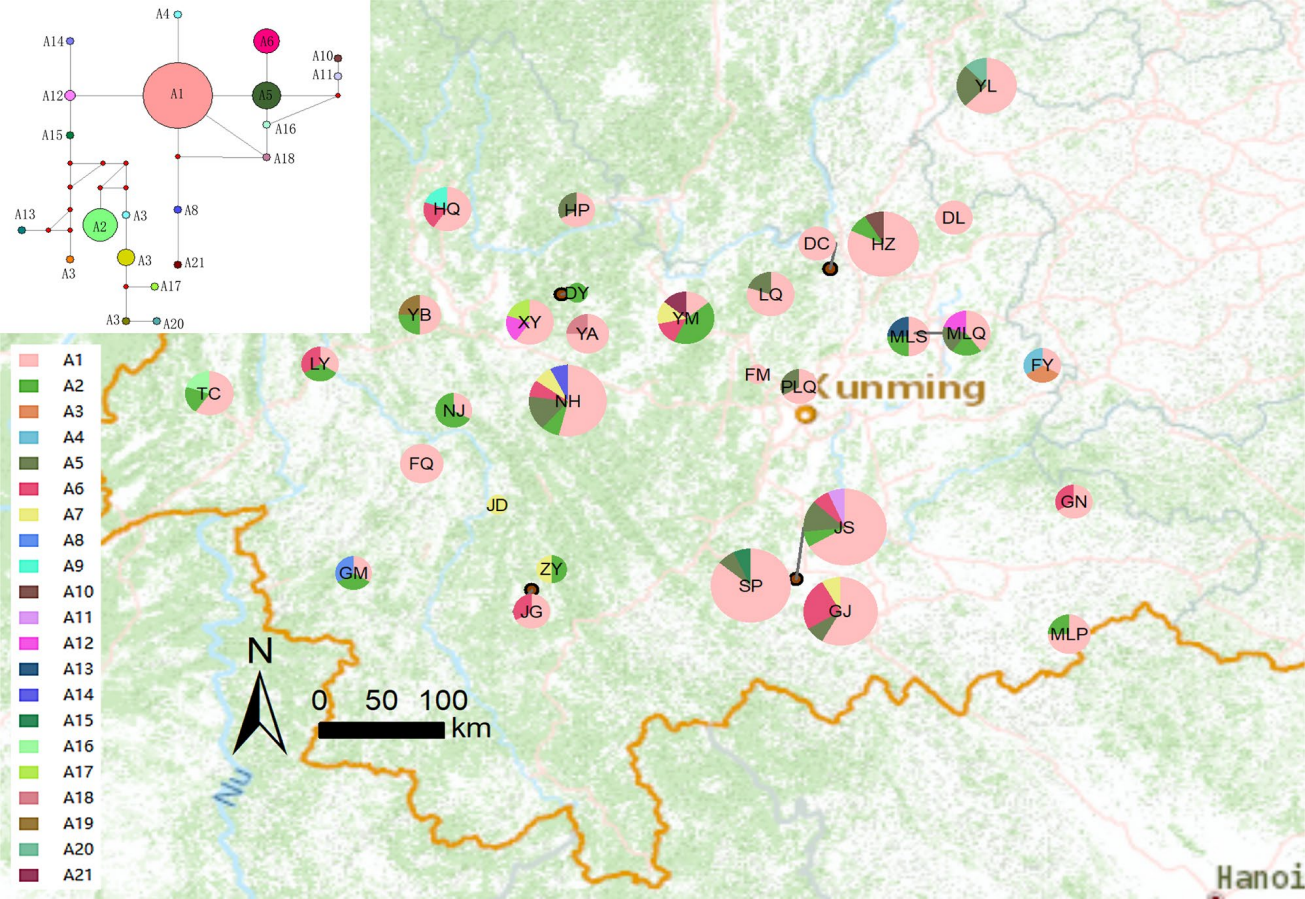
Collection locations and cp genome haplotype distribution of *Cucurbita ficifolia* germplasm resources. (**A**) Network map showed genetic analyses of 21 haplotypes. (**B**) Distribution of 21 haplotypes from 31 populations. Note: A1-A21 showed 21 haplotypes of *C. ficifolia.* The MAP is taken from CGIAR-CSI (https://srtm.csi.cgiar.org)

We sampled a total of 160 *C. ficifolia* individuals from 31 different locations, and each location contained 1–15 individuals (Fig. 1 and Supplementary Table 1), Each sampled plant was at least 50 m apart. The *C. ficifolia* sampling locations were chosen to cover the main *C. ficifolia* growing areas in China. The materials were collected from artificial planting bases or from wild-growing plants, and official collection permits were not required because this species is not included on the Chinese List of National Key Protected Plants. The plant materials were formally identified by Yongjie Guo of the Kunming Institute of Botany, based on morphological characters. A voucher specimen of *C. ficifolia* has been deposited in the herbarium of the Kunming Institute of Botany, Chinese Academy of Sciences (KUN 1580438).

Fresh leaf tissue was stored at -80°C, and total DNA was extracted leaf samples using the CTAB method [35]. 1.0% Agarose gel electrophoresis (Omega Bio-Tek, Norcross, GA, United States) and a fluorometer (Qubit3.0, Thermo Fisher Scientific, Waltham, MA, United States) were used to quantify the DNA in each sample and assess its quality. DNA samples of sufficient quality were standardized to the same volume (10 µl) and quantity of DNA (200 µg). The Illumina NovaSeq6000 sequencing platform was used to randomly fragment the genomic DNA. Libraries were constructed from randomly fragmented genomic DNA (insert sizes ∼450 base-pairs (bp)), and 150 bp paired-end reads were generated. The raw sequencing data were filtered using fastp 0.21.0 with the parameters “fastp -q 10 -u 50 -y -g -Y 10 -e 20 -l 100 -b 150 -B 150”. Low-quality reads (50% or more of the bases with a quality score < 10) and poly-Ns (10% or more of the bases were Ns) were filtered out. Low-quality bases (Q ≤ 13) were removed, and adapters were removed from both ends. The clean data were used in the subsequent analyses.

### Chloroplast genome assembly, gene annotation and sequence analysis

The clean reads were assembled using the GetOrganelle pipeline (https://github.com/Kinggerm/GetOrganelle). A reference genome *C. moschata* (Duch. ex Lam.) Duch. ex Poiret [36] was used to check the contigs, using BLAST (https://blast.ncbi.nlm.nih.gov/); the contigs were then aligned and oriented according to the reference genome. Annotation of the genome was automatic using the CpGAVAS pipeline [37] and Geneious 8.1 [38] was used to adjust the start/stop codons and intron/exon boundaries. The tRNA was identified using tRNAscan-SE v2.0 [39]. A physical map of the cp genome was generated using the online tool OGDraw v1.2 (http://ogdraw.mpimp-golm.mpg.de/) [40].

### Analysis of the features of the chloroplast genome

Chloroplast genomes contain repetitive sequences, which are believed to be important in genome rearrangement and stabilization [41]. REPuter [42] was used to find forward tandem repeats, reverse repeats, complement repeats and palindromic repeats ≧ 16 bp in the cp genome of *C. ficifolia*, with a minimum alignment scored and maximum period size of 500. SSR markers in the genomes were identified using Phobos v3.3.12 [43] and SSRHunter [44], which use a recursive algorithm to identify dinucleotide and other multinucleotide repeats with lengths between two and six base pairs with at least four copies. Analysis of codon usage and calculation of relative synonymous codon usage (RSCU) were conducted using the MEGA v11 software [45].

### Chloroplast genome genetic diversity analyses based on 160 individuals

For the identification of *C. ficifolia* varieties, we used MAFFT V7.471 (Kazutaka Katoh, Japan) which resulted in an alignment data matrix that could be used for DNAsp analysis [46]. Insertion/deletion polymorphisms (indels) and single nucleotide polymorphisms (SNPs) in the cp genome were then identified using DNAsp [47]. All indels found in the aligned sequences were included in the following analyses. DNAsp was also used to conduct a sliding window analysis [47], where the window length was set to 100 bp and the step size to 25 bp. Haplotype data files were generated in DNAsp and the haplotype diversity (*H*_*d*_) was also calculated.

### Structure of the *C. ficifolia* genome and comparison of the genome with others from the genus *Cucurbita*

Although the IRs are highly conserved in cp genomes, contraction and expansion at their borders are common in evolutionary time, and may significantly influence their boundaries with the LSC or SSC regions, as well as leading to size variations in different cp genomes [48–50] To compare the IR boundaries in several *Cucurbita* species, the cp genomes of seven *Cucurbita* species were downloaded from NCBI and compared with the *C. ficifolia* cp genome using IRscope10 [51].

Comparative analysis of different cp genomes is extremely important in genomics [52]. We used the online software mVISTA11 [53] using the Shuffle-LAGAN alignment model [54] to determine the differences between the cp genomes of the seven study *Cucurbita* species, with *C. argyrosperma* as a reference.

### Phylogenetic reconstruction and population structure analysis

The cp genome sequences of 61 Cucurbitaceae species as well as an outgroup (*Lavandula angustifolia*, Lamiaceae) were downloaded from GenBank. MAFFT [46] was used to construct an alignment of the 61 downloaded sequences with the 21 *C. ficifolia* cp genome haplotype sequences from our study. To resolve the phylogenetic placement of *C. ficifolia* within the Cucurbitaceae, a maximum likelihood (ML) phylogenetic tree was reconstructed in MEGA v11 [45] using the cp genome sequences with the GTR + GAMMA substitution model and including a tree robustness assessment using 1000 replicates of rapid four bootstrap, the GTR + GAMMA model was chosen through “Find Best DNA model” in MEGA v11 [45].

## Results

### Characteristics of the *C. Ficifolia* chloroplast genome

**Fig. 2:**
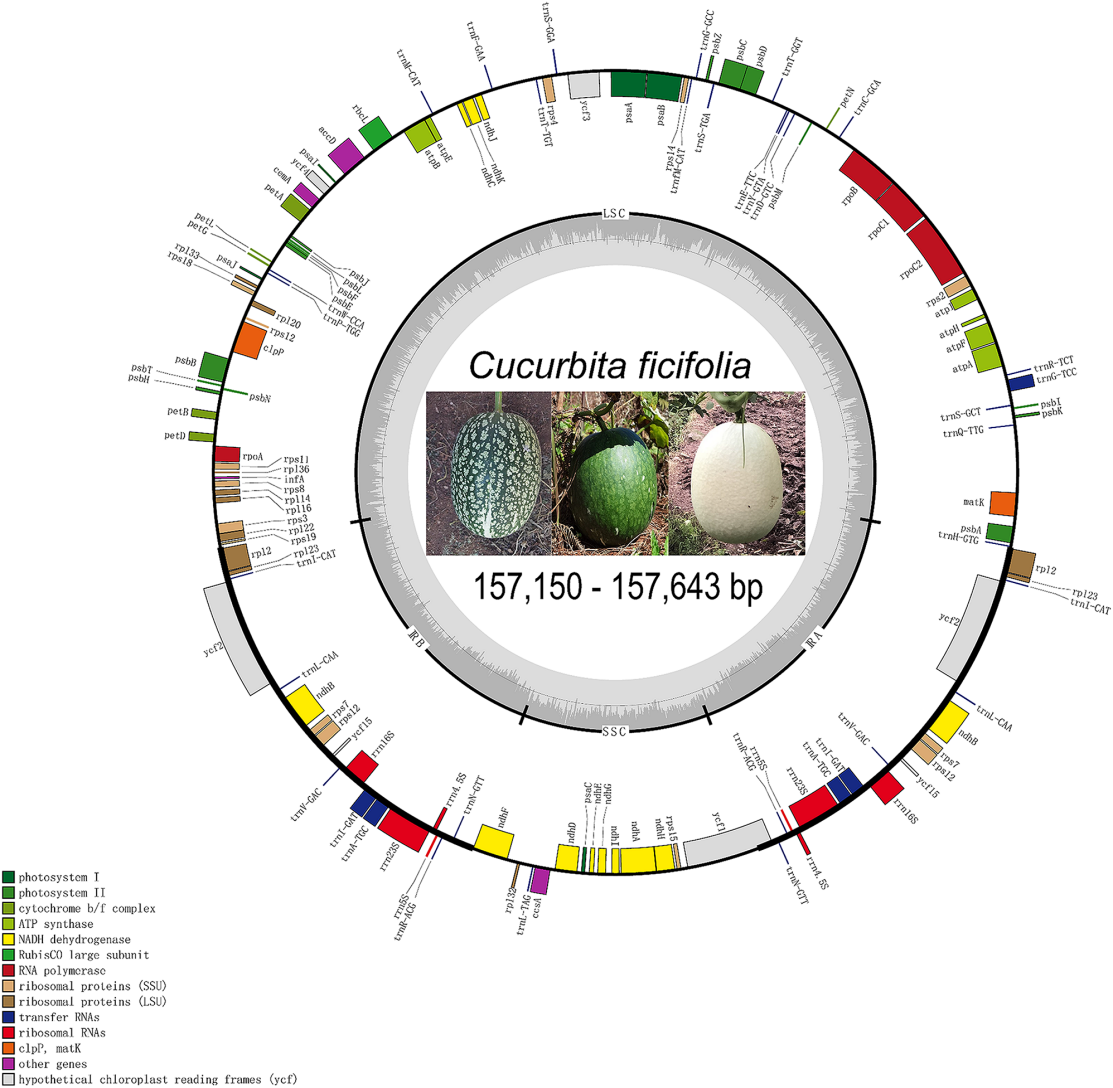
Gene map of the *Cucurbita ficifolia* chloroplast genome

The collection localities of the 160 *C. ficifolia* study individuals are shown in Fig. 1. Resequencing these 160 individuals on an Illumina NovaSeq6000 sequencer generated 758.08 Gbp of clean data, with a total of 2.2 million 100 bp paired-end reads (332 Gb of sequence data), 93.63% of which had a Q value ≥ 30. The average rate of alignment of samples to the reference genome was 93.17%, the average depth of coverage was 10 × and the genome coverage was 66.11% (with at least one base coverage). The above resequencing data were then used to assemble and annotate the complete cp genome of *C. ficifolia*. We found that the *C. ficifolia* cp genome was circular and double-stranded, and that it ranged in size in our study individuals from 157,150 to 157,643 bp (Fig. 2). The genome comprised the LSC (87,730 − 88,210 bp), the SSC (18,136 − 18,144 bp), and IRa and IRb (25,638 − 25,597 bp). Throughout the genome, the GC content was 37.2% on average, with the IRa, IRb, SSC and LSC having 43.0, 43.0, 31.6 and 34.9% GC content, respectively (Table 1).

**Table 1:** Base composition of *Cucurbita ficifolia* cp genomic regions

Region	T (%)	C (%)	A (%)	G (%)	GC (%)	Length (bp)
LSC	33.4	17.8	31.7	17.1	34.9	87,730–88,210
SSC	34.2	16.6	34.2	15.0	31.6	18,136–18,144
IRa	28.5	22.3	28.5	20.7	43.0	25,638 − 25,597
IRb	28.5	22.3	28.5	20.7	43.0	25,638 − 25,597
Total	31.9	18.8	31.0	18.3	37.2	157,150–157,629

*Note LSC* large single-copy regions; *SSC* small single-copy regions; *IRa* inverted repeat A; *IRb* inverted repeat B

The *C. ficifolia* cp genome contained 128 genes in all individuals except one (FY_H2), where the *ycf2* of FY_H2 was unannotated due to the presence of multiple termination codons. The *atpA* genes in individuals FY_H1, YB_H2 and YL_W1 were terminated prematurely and *atpA* was annotated as a pseudogene. The remaining individuals were all found to have 86 protein-coding genes (PCGs), 34 transfer RNA (tRNA) genes and 8 ribosomal RNA (rRNA) genes (Fig. 2). These genes were divided into three functional categories: photosynthesis (47 genes), self-replication (70), biosynthesis (6) and genes of unknown function (5) (Fig. 2; Table 2). In the IR regions, nineteen gene species were duplicated either completely or partially, including eight PCGs, (*ndhB*, *ndhF*, *rpl2*, *rpl23*, *rps7*, *rps12*, *ycf2* and *ycf15*), seven genes encoding tRNAs (*trnA-TGC*, *trnI-CAT*, *trnI-GAT*, *trnL-CAA, trnN-GTT*, *trnR-ACG* and *trnV-GAC*), and the four genes encoding rRNAs (4.5 S, 5 S, 16 S and 23 S). The results of gene structure analysis suggested that the *C. ficifolia* genome included seven genes that contained introns, of which four were found in the LSC and one in the SSC (*ndhA*). Five genes contained a single intron (*atpF*, *ropC1*, *ycf3*, *ndhA*, *ndhB*) and two contained two introns (*clpP* and *rpl2*) (Table 3). The structural elements were almost identical between the 160 *C. ficifolia* varieties, suggesting that the structure of the cp genome is highly conserved in this species.

**Table 2:** Genes present in the *Cucurbita ficifolia* chloroplast genome

Category	Gene groups	Name of genes
Self-replication	Large subunit of ribosomal proteins	*rpl14, rpl16, rpl2*^*2*^, *rpl20, rpl22, rpl23*^*2*^, *rpl32, rpl33, rpl36*
	Small subunit of ribosomal proteins	*rps2, rps3, rps4, rps7*^2^, *rps8, rps11, rps12*^*2*^, *rps14, rps15, rps18, rps19*
	DNA dependent RNA polymerase	*rpoA, rpoB, rpoC1, rpoC2*
	Ribosomal RNA genes	*rrn4.5*^*2*^, *rrn5*^*2*^, *rrn16*^*2*^, *rrn23*^*2*^
	Transfer RNA genes	*trnA(TGC)*^2^, *trnC(GCA)*, *trnD(GTC)*, *trnE(TTC)*, *trnF(GAA)*, *trnfM(CAT)*, *trnG(GCC)*, *trnG(TCC)*, *trnH(GTG)*, *trnI(CAT)*^2^, *trnI(GAT)*^2^, *trnL(CAA)*^2^, *trnL(TAG)*, *trnM(CAT)*, *trnN(GTT)*^2^, *trnP(TGG)*, *trnQ(TTG)*, *trnR(ACG)*^2^, *trnR(TCT)*, *trnS(GCT)*, *trnS(GGA)*, *trnS(TGA)*, *trnT(GGT)*, *trnT(TGT)*, *trnV(GAC)*^2^, *trnW(CCA)*, *trnY(GTA)*
Photosynthesis	NADH oxidoreductase	*ndhA, ndhB*^2^, *ndhC, ndhD, ndhE, ndhF, ndhG, ndhH, ndhI, ndhJ, ndhK*
	Photosystem I	*psaA, psaB, psaC, psaI, psaJ, ycf3, ycf4*
	Photosystem II	*psbA, psbB, psbC, psbD, psbE, psbF, psbH, psbI, psbJ, psbK, psbL, psbM, psbN, psbT, psbZ*
	Cytochrome b/f complex	*petA, petB, petD, petG, petL, petN*
	ATP synthase	*atpA, atpB, atpE, atpF, atpH, atpI*
	RubisCo large subunit	*rbcL*
Other genes	Maturase K	*matK, cemA*
	C-type cytochrome synthesis gene	*ccsA*
	Protease	*clpP*
	Translational initiation factor	*infA*
	Subunit of cetyl-CoA-carboxylase	*accD*
	Proteins Proteins of unknown function	*ycf1, ycf2*^2^, *ycf15*^2^

*Note *^2^Two gene copies in IRs

**Table 3:** The lengths of exons and introns in intron-containing genes of the *Cucurbita ficifolia* cp genome

Gene	Location	Size (bp)	Exon (bp)	Intron (bp)	Exon (bp)	Intron (bp)	Exon (bp)
atpF	LSC	1306	410	748	148		
rpoC1	LSC	2798	1611	752	435		
ycf3	LSC	1989	153	749	230	730	127
clpP	LSC	2036	228	600	294	845	69
rpl2	IR	1490	470	629	391		
ndhB	IR	2219	756	671	792		
ndhA	SSC	2233	539	1138	556		

### Chloroplast genome genetic diversity analyses based on 160 individuals

**Fig. 3:**
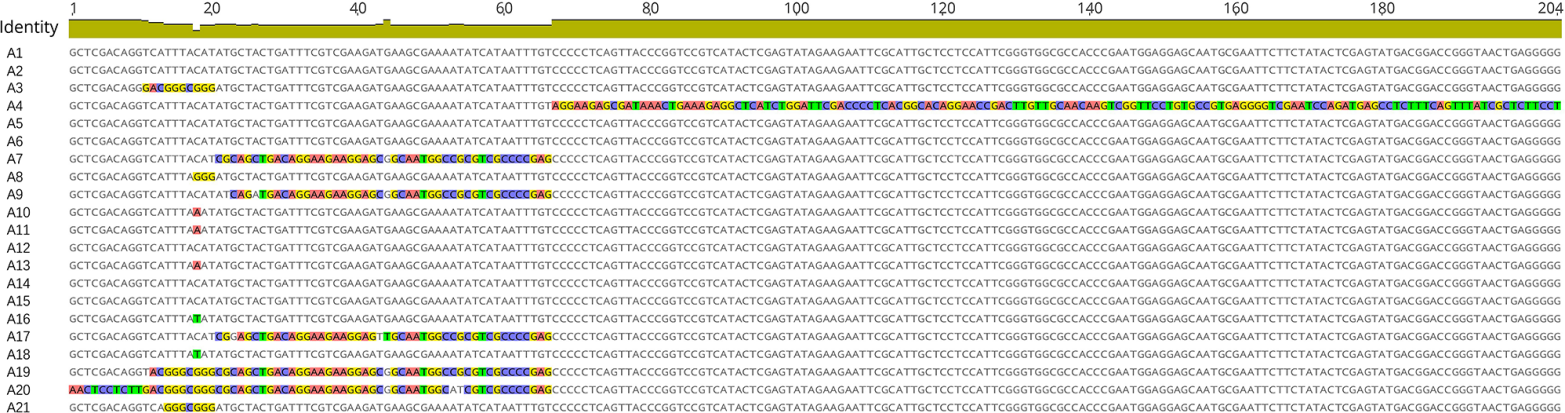
Polymorphic sites in the chloroplast (cp) genomes of 21 *Cucurbita ficifolia* haplotypes

**Fig. 4:**
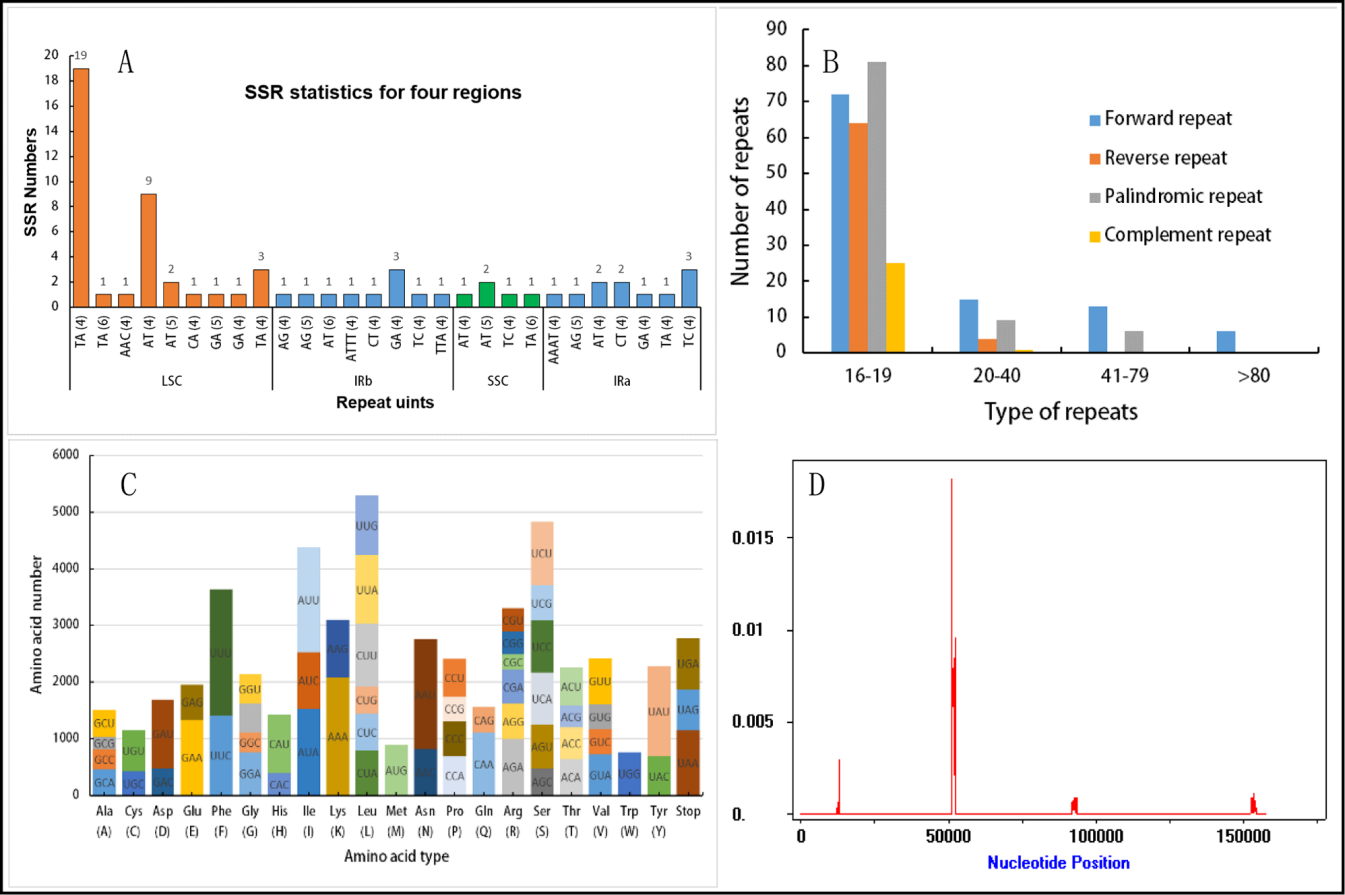
Types and distributions of repeat sequences and short sequence repeats (SSRs) in *Cucurbita ficifolia* chloroplast (cp) genomes. (**A**) Proportion of SSRs in *C. ficifolia* cp genomes. (**B**) Numbers of different types of repeat sequences in the *C. ficifolia* cp genomes. (**C**) Codon content for the 20 amino acids and stop codons in 86 protein-coding genes of *C. ficifolia* chloroplast genomes. (**D**) Sliding window analysis of 160 complete chloroplast (cp) genomes from *Cucurbita ficifolia*. The x-axis represents the midpoint of the window and the y-axis represents the nucleotide diversity (Pi) of each window. The window length is 600 bp with a 200-bp step size

A total of 57 indels and 204 SNPs were found in the data matrix of our 160 individuals. Of the 204 SNPs, 149 were singleton variable sites and 55 were parsimony-informative sites (Fig. 3). A total of 21 haplotypes were resolved in the 160 sample *C. ficifolia* individuals and the haplotype diversity (*Hd*) was 0.598. Haplotype A1 was most widespread, appearing in 99 individuals from 28 populations, followed by A2, which appeared in 13 populations. 15 haplotypes occurred only once (Fig. 1). Populations JS and NH harbored five haplotypes and GJ had four. Most other populations had only one or two haplotypes. From the network analysis, the dominant haplotype A1 could form haplotypes A4, A5 and A12 through a single mutation, and A8 through two single mutations. The 21 haplotypes formed a network model, but not a linear model, meaning that they have a complex evolutionary relationship (Fig. 1). The sliding window analysis of 21 haplotypes showed that most variation occurred in four regions, especially around the position of 50,000 bp (Fig. 4A).

### SSRs, repeat sequences and codon usage bias in the *C. Ficifolia* chloroplast genome

Because A1 was the most widespread haplotype among the 160 *C. ficifolia* individuals, it was chosen for the following SSR, repeat and codon usage bias analyses. The cp genome of *C. ficifolia* haplotype A1 contained only 64 identified SSRs (Fig. 4A). There were 19 SSRs of type TA (4), and 9 of type AT (4), and most of the SSRs appeared only once. Dinucleotide, trinucleotide and tetranucleotide SSRs represented 99.4, 0.03, and 0.03% of the total SSRs, respectively (Fig. 4B). The LSC region contained the great majority of the SSRs (59.3%). Ten SSRs were found in the IR regions, and only five were found in the SSC. A/T repeats represented 70.3% of the *C. ficifolia* cp SSRs, indicating that there was an A/T nucleotide bias.

We then analyzed four types of repetitive sequences: forward, reverse, palindromic, and complement repeats. The *C. ficifolia* cp haplotype A1 contained 296 repeat sequences, with 106, 68, 96 and 26 forward, reverse, palindromic and complement repeats, respectively, which ranged in length from 16 to 200 bp, with most (accounting for 80.1% of the total) being 16–20 bp. A palindromic repeat in the LSC region was the longest at 166 bp. The locations of the repeats are given in Fig. 4C.

The protein-coding genes were then analyzed for codon usage. We found 45 codons with an RSCU > 1.0. The five most commonly used codons were UUU (4.24%), AAA (3.97%), AAU (3.69%), AUU (3.50%) and UAU (3.04%). The most common amino acids were Leu (L), Ser (S), Ile (I), all of which occurred > 4000 times. Conversely, the amino acids Met (M) and Trp (W) were used rarely, with fewer than 1000 occurrences (Fig. 4D). Codon preference analysis results showed that the 3’ ends of most codons, containing A or T, had RSCU values higher than 1, and that these codons were preferred.

### IR expansion and contraction in the *Cucurbita* cp Genome

**Fig. 5:**
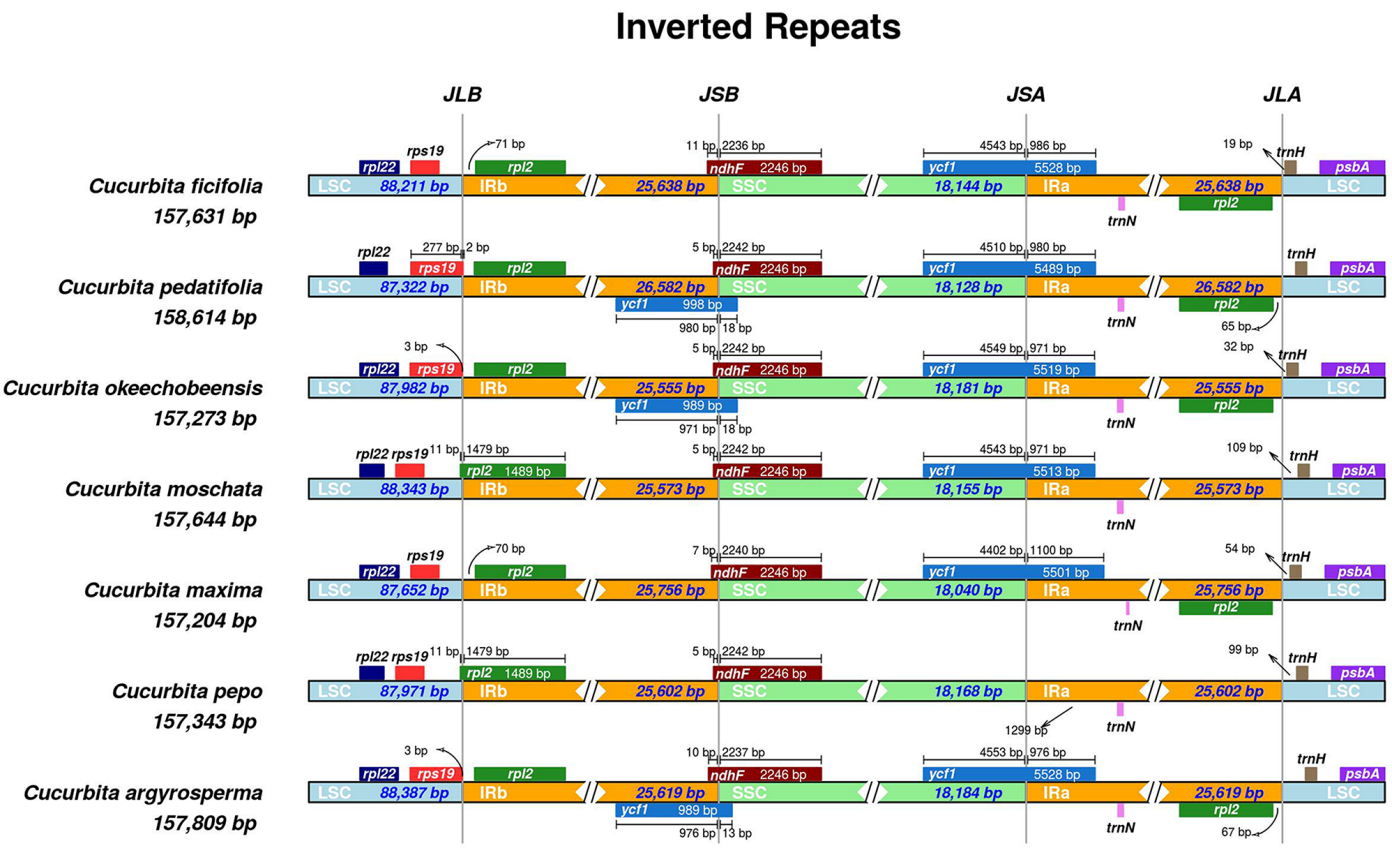
Comparison of border distance between adjacent genes and junctions of the LSC, SSC and two IR regions among the chloroplast genomes of seven *Cucurbita* species. Boxes above or below the main line indicate the adjacent border genes. The figure is not to scale with respect to sequence length, and only shows relative changes at or near the IR/SC borders

We then compared the IR boundaries characteristic of *C. ficifolia* cp genomes of haplotype A1 to the cp genomes of six other *Cucurbita* species (*C. argyrosperma*, *C. maxima*, *C. moschata*, *C. okeechobeensis*, *C. pedatifolia*, *C. pepo*). The complete cp genomes of these *Cucurbita* species ranged in length from 157,204 bp (*C. maxima*) to 158,614 bp (*C. pedatifolia*). All of the cp genomes included in this study had a structure typical of the angiosperms, being quadripartite and including a large and a small single-copy region, and two inverted repeat regions (Fig. 5; Table 4). We compared the genomic regions spanning the IR/LSC and IR/SSC junctions in our seven study species. The length of IR regions ranged from 25,555 in *C. okeechobeenisis*, which also had the smallest cp genome, to 26,582 bp in *C. pedatifolia*, which had the largest cp genome. Similarly, the LSC regions ranged in length from 87, 322 in *C. pedatifolia*, which also had the smallest cp genome, to 88, 387 bp in *C. argyrosperma*, which had the largest cp genome. There was no significant difference in the size of the SSC among these four species, and variation in the sizes of the IR and LSC regions appears to be the main reason for the differences in length seen in the different *C. ficifolia* cp genomes.

**Table 4:** Comparison of the features of the *Cucurbita* species chloroplast genomes

Species	Genome Size (bp)	LSC size (bp)	SSC size (bp)	IR Size (bp)	Genome GC (%)	Total gene	Protein coding genes	rRNA genes	tRNA genes	Total unique genes
*C. ficifolia*	157,631	88,211	18,144	25,638	37.2	129	87	8	34	
*C. pedatifolia*	158,614				37.1	129	85	8	36	
*C. okeechobeensis*	157,273	87,982	18,181	25,555	37.1	129	85	8	36	
*C. moschata*	157,644				37.1	135	85	8	42	
*C. maxima*	157,204				37.1	130	85	8	37	
*C. pepo*	157,343				37.2	129	84	8	37	
*C. argyrosperma*	157,809	88,387	18,184	25,619	37.1	129	85	8	36	

We found a few differences in the IR/LSC and IR/SSC junction regions among our study species. Five genes were present at the IR/LSC or IR/SSC boundaries: *rps19*, *rpl2*, *ycf1*, *ndhF* and *trnH*. We then analyzed the characteristics of the four boundaries IRa-SSC (JSA), IRa-LSC (JLA), IRb-LSC (JLB), and IRbSSC (JSB). We found that the JLB boundary lay in the intergenic region between *rps19* and *rpl2* in four *Cucurbita* cp genomes, but was located in the *rpl2* gene in *C. moschata* and *C. pepo*, and in *rps19* for *C. pedatifolia*. The JSB boundary was consistent throughout all the tested species and was located in the *ndhF* gene, and the JSA boundary was located in the *ycf1* gene. The JLA boundary was located between the *rpl2* and *trnH* genes (Fig. 5). The IR/LSC and IR/SSC junction regions are therefore relatively conserved between different *Cucurbita* species.

### Comparative analysis of chloroplast genomes in Cucurbita species using mVISTA

**Fig. 6:**
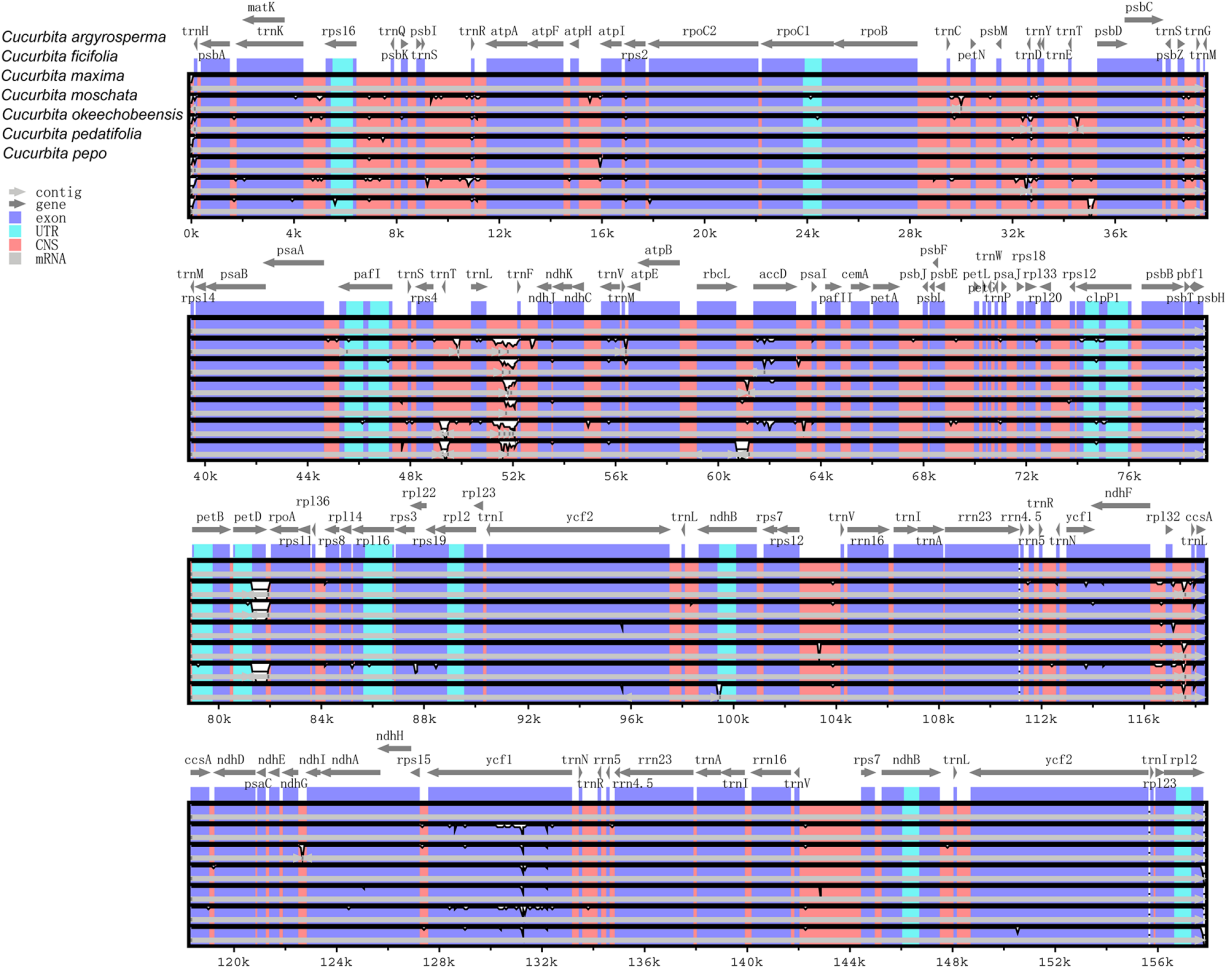
Comparison of four cp genomes using the mVISTA alignment program. The x-axis represents the coordinates in the cp genome. The y-axis indicates the average percent identity of sequence similarity in the aligned regions, ranging between 50% and 100%. Purple bars represent exons, blue bars represent untranslated regions (UTRs), pink bars represent noncoding sequences (CNS), gray bars represent mRNA, and white bars represent differences in genomics

Multiple alignments of the cp genomes from our seven study *Cucurbita* species were constructed in the mVISTA software, using *C. argyrosperma* as a reference (Fig. 6). Overall, the sequences of the cp genomes in *Cucurbita* species were highly conserved. Unsurprisingly, we found that the coding regions were more highly conserved than non-coding regions, and that the IR regions were less divergent than the LSC and SSC regions. Notably, intron-containing genes were found to have high levels of variability.

The intergenetic spacers, including *trnL-trnF*, *trnT-trnL*, *rpl32-trnL*, *rbcL-accD*, *trnS-trnR*, *rps12-trnV* were the most highly divergent sequences in the seven cp genomes studied. The coding regions with the highest divergence were the *accD*, *petD*, *ycf1* and *ycf2* sequences. This is similar to the results obtained in previous studies [55–57], and suggest that these regions might evolve rapidly in *Cucurbita*, and could therefore be useful in the identification of *Cucurbita* species.

**Fig. 7:**
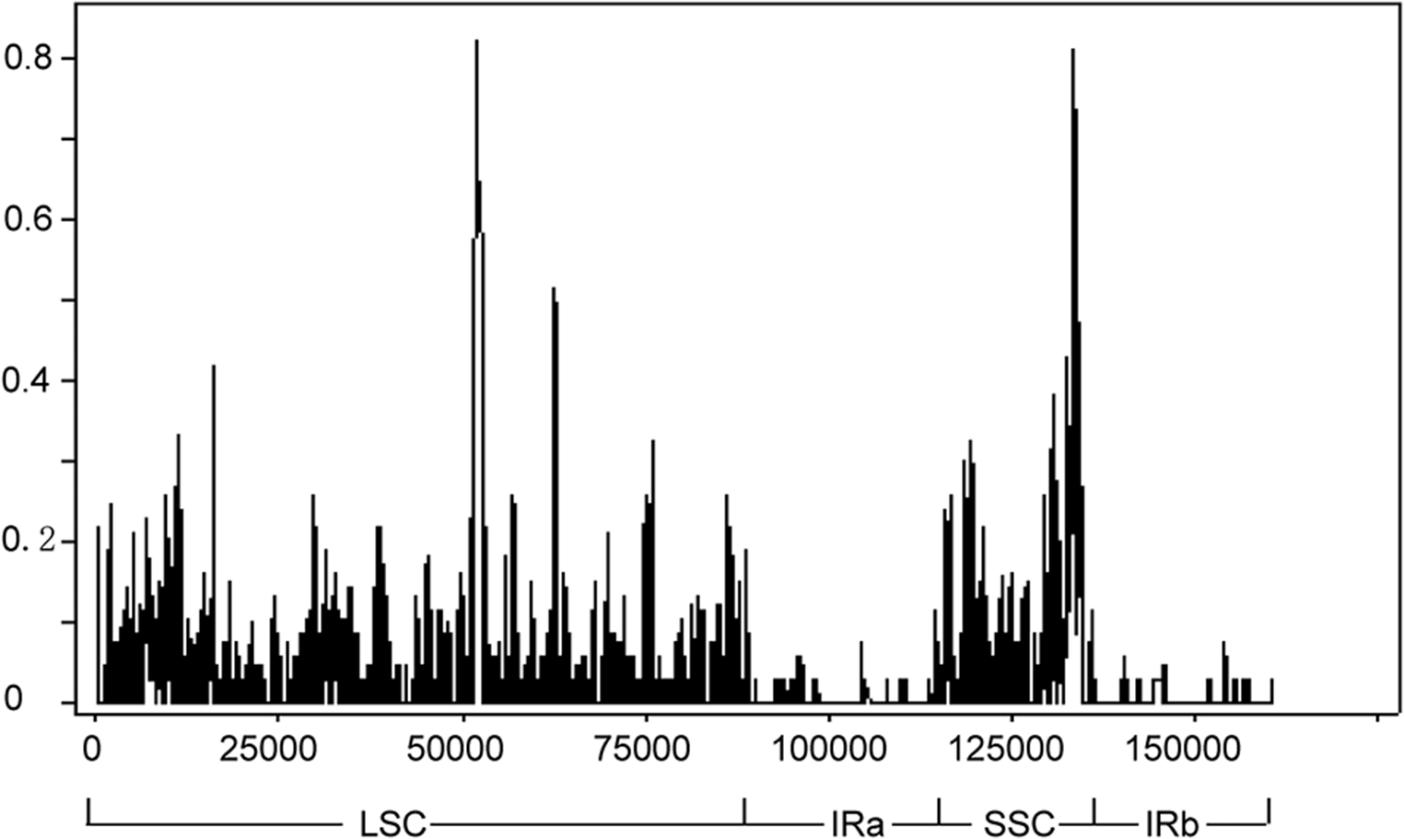
Sliding window analysis of the complete chloroplast (cp) genomes from seven different *Cucurbita* species. The x-axis represents the midpoint of the window and the y-axis represents the nucleotide diversity (Pi) of each window. The window length is 600 bp with a 200-bp step size

We used DNAsp to investigate nucleotide variability (π) and levels of sequence divergence within the aligned genome sequences from the seven study species. The nucleotide variability (π) was found to be 0.0034, showing that the genomes were relatively divergent despite the relatedness of the study species. 1,486 SNPs were found. The sliding window analysis of this genus showed that most variation occurred in the LSC and SSC regions, with the IR region being relatively conservative (Fig. 7). Our results suggest that the cp genome could informative for the reconstruction of species-level phylogenies this plant group, and that the LSC and SSC regions are a good choice when searching for loci for genetic diversity and phylogenetic analyses.

### Phylogenetic analysis of 61 Cucurbitaceae species and 21 haplotypes of *Cucurbita*

**Fig. 8:**
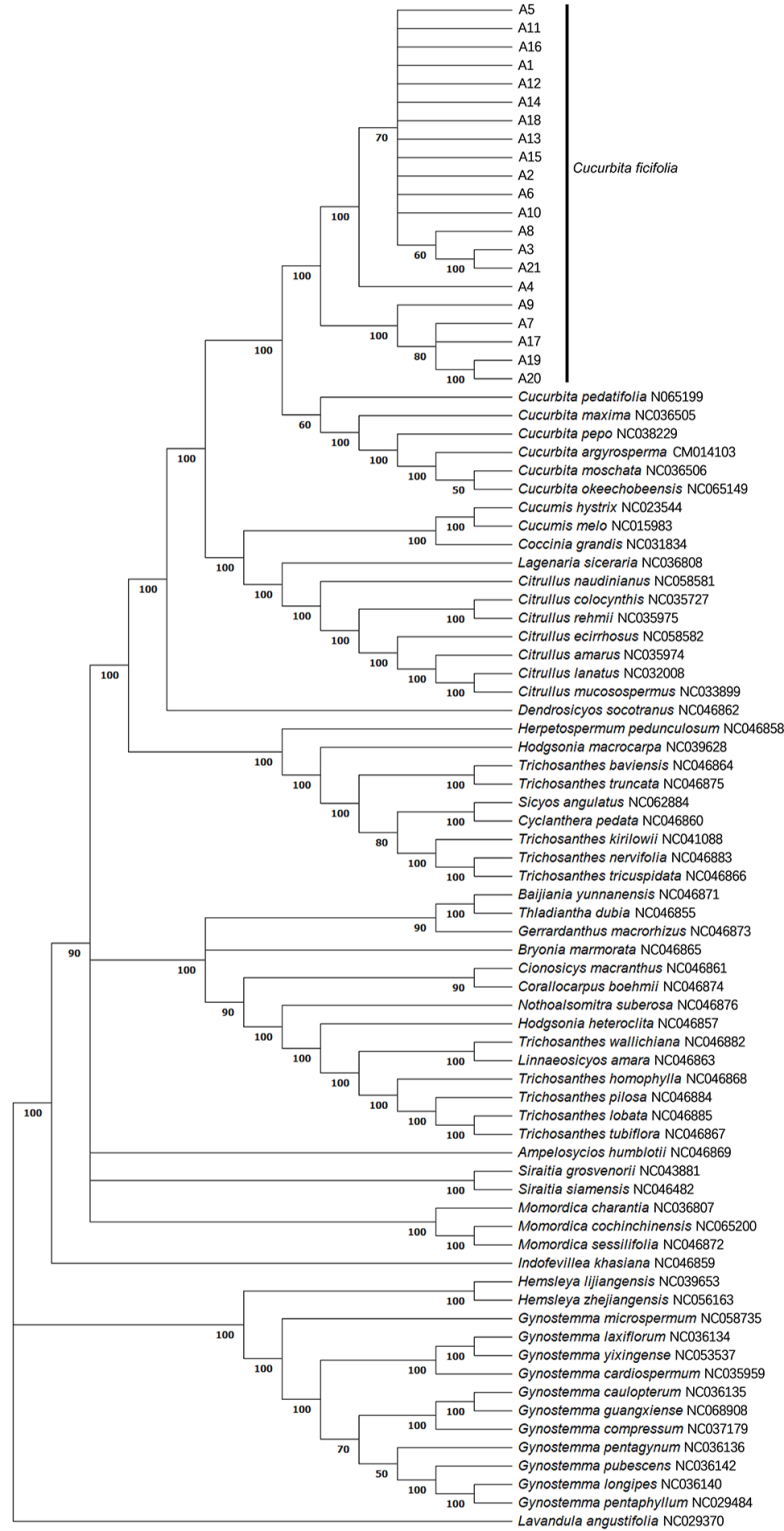
Reconstructed maximum likelihood (ML) phylogenetic tree based on the chloroplast genome sequences of different species of Cucurbitaceae. *Lavendula angustifolia* (Lamiaceae) was used as an outgroup. Numbers to the right of nodes are bootstrap support values

To explore the evolutionary relationships among Cucurbitaceae species, the 21 cp genome haplotypes identified in *C. ficifolia* as well as the cp genome sequences of 61 other species in the Cucurbitaceae were aligned and used to reconstruct a phylogeny (Fig. 8). *Lavandula angustifolia* (Lamiaceae) was selected as an outgroup. ML trees were constructed using the whole cp genome. The different genera within the Cucurbitaceae can be distinguished in the phylogeny, meaning that the phylogeny reconstructed from cp genomic data was consistent with the traditional classification of this group. The *Cucurbita* species demonstrated a close genetic relationship and clustered together in a single branch. The 21 haplotypes identified from *C. ficifolia* also clustered together, reflecting the close evolutionary relationships among the different ecotypes of the same species. These results indicate that the whole cp genome is a reasonable choice for investigation of the evolutionary relationships within the Cucurbitaceae.

## Discussion

### Characteristics of the C. ficifolia *chloroplast genome*

The entire cp genome of *C. ficifolia* showed a conserved quadripartite structure. The length of the two reverse repeat regions was similar to those in most terrestrial plants. The IR region contained the rRNA genes, and had a lower GC content than that of the LSC and SSC regions. Overall, the cp genome had an AT content higher than the GC content, which reflects results reported from the chloroplast genomes from most higher plants [58]. A total of 7 genes in the *C. ficifolia* cp genome were found to contain introns. We then classified the cp genome of *C. ficifolia* using gene annotation, and divided the genes into three major categories according to function: genes for the photosynthetic system, genes for the genetic system and open reading frame and other genes. The results are basically consistent with those reported from other plants in *Cucurbita* [59, 60]. Codon preference analysis showed that the RSCU values of the 3’ ends of most codons containing A or T were higher than 1, and that these codons were preferred. We speculated that this might be due to the fact that the AT content of the whole *C. ficifolia* cp genome was enriched. Previous studies have shown that the second codon also has an AT bias [61, 62].

Repetitive sequences are widely found in cp genomes in higher plants and are an important source of genome variation [63]. The *C. ficifolia* cp genome contains 296 tandem repeats, suggesting that these repeats might lead to recombination or rearrangement of the cp genome during its evolution. Simple sequence repeats (SSR) are widely used as DNA markers [64]. We detected 67% of SSR markers in the *C. ficifolia* cp genome were found in the LSC and SSC regions, and a few in the IRs. This agrees with the results of many studies into the cp genome. We speculate that this number may be due to the repetitive nature of the IRs, which leads to sequence duplication and correction. In *C. ficifolia*, the cp genomic sequence has A/T base bias, and the SSR sequences mainly comprise poly-adenine (poly A) and poly-thymine (poly T) runs, which is consistent with our previous analysis of the *C. ficifolia* cp genome sequence. Chloroplast SSRs can be useful in phylogenetic analyses and species identification as well as in the study of species evolution and variation [65] and the SSRs detected in the *C. ficifolia* cp genome will therefore be important in future phylogenetic and population genetics studies in *Cucurbita* and the Cucurbitaceae.

### Differences in the chloroplast genome and genetic diversity in C. ficifolia *landraces*

Several species from the genus *Cucurbita* are important as vegetables, and many different local cultivars and landraces have been developed [2]. Seed exchange has led to germplasm selection, and natural and artificial hybridization has contributed to genetic variation [66]. Collections of germplasm resources from different cultivars and landraces therefore represent a wide range of genetic diversity, which is of interest in the development of new cultivars with particular characteristics [67]. However, extensive genetic diversity studies are necessary before these germplasm resources can be effectively used. We sequenced the cp genomes of 160 individuals of *C. ficifolia* to investigate genetic diversity in this species. The haplotype diversity (*Hd*) was a little high as a domesticated specie. This could mean that *C. ficifolia* developed new genetic diversity in order to adapt to the local climate after its spread from the Central-South American region [12] and these new mutations represent important germplasm for the utilization of *C. ficifolia.* Network modeling of haplotypes showed complex genetic relationships within the *C. ficifolia* genetic resources. The variable geography and climate of southwest China may be the driving force behind this genetic variation, and anthropogenic intervention on the genetic structure of this species should also be considered.

### Comparison of the chloroplast genomes of different Cucurbita *species provides new insights into the phylogeny of this genus*

We compared the cp genome sequences of seven species of *Cucurbita*. The genomes ranged in size from 157,204 bp (*C. maxima*) to 158,614 bp (*C. pedatifolia*), and all showed a conserved tetrad ring structure, consistent with cp genomes from other higher plants [68, 69]. We speculate that the different lengths of chloroplast genomic regions in *Cucurbita* is a result of shrinkage or expansion of the IR region compared to other Cucurbitaceae cp genomes [70–72]. IR regions shrinkage or expansion is relatively common [73, 74]. We found that SSC/IRa, SSC/IRb and LSC/IRa and had similar gene distribution patterns, and the ycf1 gene spanned the border between IRa and SSC. The rpl22 gene was located upstream of the LSC-IRa junction, and the rp12 gene overlapped the LSC-IRb region. This subtle span length can also be applied to species classification.

The maternally inherited cp genome evolves independently from the nuclear genome. It is also small and easy to isolate and sequence. These factors, as well as the moderate rate of base variation, means that the cp genome is often used as the basis for the study of phylogenetic relationships. The Cucurbitaceae family comprises about 1,000 species, and economically important plants in this family are widely cultivated in low latitudes with warm climates [75]. In order to reveal the phylogenetic relationships within the genus *Cucurbita* and their phylogenetic relationships with other species of *Cucurbita*, the cp genomes of 21 haplotypes of *C. ficifolia*, 6 species of *Cucurbita* and 56 further species from the Cucurbitaceae were selected as a data set with which to construct a phylogenetic tree. The 21 haplotypes of *C. ficifolia* formed a monophyletic group, and the six other species of *Cucurbita* clustered together. Schaefer et al. [76] investigated the history of the Cucurbitaceae using a multigene phylogeny for 114 species, and found that *Cucurbita* spp. have an apparent Central or South American origin, and that the split of the genus from its sister clade, *Peponopsis*, occurred about 16 (23 − 9) Myr ago. Chomicki et al. (2020) studied the phylogenetic distribution of cultivated Cucurbitaceae and made estimations of ancestral state on a phylogeny sampling 554 Cucurbitaceae species. The results suggested that the genus *Cucurbita* has a close relationship with *Cucumis*, *Coccinia*, *Lagenaria* and *Citrullus*. This close relationship is also indicated in our study.

In our study, the clade formed by *C. ficifolia* was sister to that comprising the rest of the *Cucurbita* species. This is consistent with the results from Kates et al. [77], Zheng et al. [78] and Sanjur et al. [79], who built phylogenies based on introns of single-copy nuclear genes, chloroplast and mitochondrial gene, respectively. However, the species-level topology of *C. argyrosperma*, *C. maxima C. moschata*, *C. okeechobeensis* and *C. pepo* in the phylogeny is obviously different when reconstructed different using different molecular markers (Fig. S1). Only one relationship among the six species in the clades remained consistent across all the previous molecular phylogenetic studies conducted to date: *C. moschata* is sister to *C. argryosperma.* However, using the whole cp genome, we found that *C. moschata* was sister to *C. okeechobeensis*. To date, only one of the three plant genomes at a time has been used to reconstruct the phylogenetic relationships in *Cucurbita*. The lack of congruence among these nuclear, mitochondrial or chloroplast-based phylogenetic trees might result from a lack of phylogenetically informative characters in one or more of the trees, or perhaps from reticulate evolution [80]. The phylogenetic reconstruction of Zheng et al. (2013), which was based on four chloroplast genes, also differed from the whole cp genome tree. This suggests that although many researchers use only a small number of gene loci to construct a phylogenetic tree, these few loci represent only a small amount of the information contained in the genome, which does not represent the evolutionary history of the whole genome. This should be considered when conducting phylogenetic analyses.

The cp genome has evolved independently of the nuclear genome. Its structure is conserved, although it contains many variable sites useful for analyzing the phylogenetic relationships between plants of the genus *Cucurbita*. However, the analysis of phylogenetic relationships using cp genome sequences has certain limitations when the study objects are species that undergo extensive interspecific or intergeneric hybridization, and the results may also be affected by introgression or incomplete lineage sorting. In order to make phylogenetic studies more instructive, more samples should be collected for analysis, and genetic analyses should contain not only chloroplast, mitochondrial and nuclear genetic information, but should also be combined with a knowledge of morphology, geography and domestication history. The analysis of the cp genomes of *C. ficifolia* and related species described here will provide basic theoretical data for further studies in this genus, and the phylogeny provides new insights into the phylogenetic taxonomic position of *C. ficifolia* within the Cucurbitaceae.

### Electronic supplementary material

Below is the link to the electronic supplementary material.

Supplementary Material 1

Supplementary Material 2

## Data Availability

The sequencing data generated in this study for the 160 samples have been submitted to the NCBI Sequence Read Archive (https://www.ncbi.nlm.nih.gov/sra/?term=PRJNA924019) under the BioProject accession PRJNA924019.
