# Supplementary material for: Sequence characteristics, genetic diversity and phylogenetic analysis of the Cucurbita ficifolia (Cucurbitaceae) chloroplasts genome
Source: BMC Genomics. 2024 Apr 18;25:384. doi: 10.1186/s12864-024-10278-2 (PMC11027378; doi:10.1186/s12864-024-10278-2)
Supplement: Supplementary file 2 — Supplementary Material 2 [file 12864_2024_10278_MOESM2_ESM.docx]

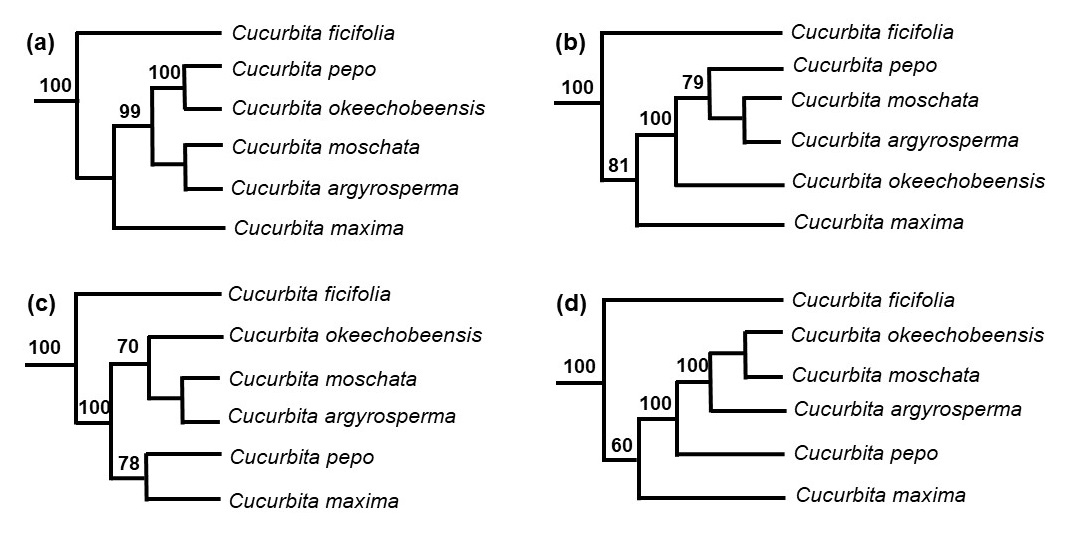


Fig. S1 Comparison of alternative branching patterns among six studied *Cucurbita* species in four phylogenetic reconstructions of *Cucurbita*. (a) Kate et al 2017 (nuclear loci); (b) Sanjur et al. (2002) (mitochondrial gene); (c) Zheng et al. (2013) (chloroplast loci); (d) The topology found in this study (cp genome).
